# Phenotypic and Genotypic Characteristic of Invasive Pneumococcal Isolates from Both Children and Adult Patients from a Multicenter Surveillance in China 2005–2011

**DOI:** 10.1371/journal.pone.0082361

**Published:** 2013-12-11

**Authors:** Chunjiang Zhao, Feifei Zhang, Yunzhuo Chu, Yong Liu, Bin Cao, Minjun Chen, Yunsong Yu, Kang Liao, Liyan Zhang, Ziyong Sun, Bijie Hu, Jin’e Lei, Zhidong Hu, Xiaobing Zhang, Hui Wang

**Affiliations:** 1 Department of Clinical Laboratory, Peking University People’s Hospital, Beijing, China; 2 Department of Clinical Laboratory, the First Hospital of China Medical University, Shenyang, Liaoning, China; 3 Department of Clinical Laboratory, Shengjing Hospital of China Medical University, Shenyang, Liaoning, China; 4 Department of Infectious Diseases and Clinical Microbiology, Beijing Chao-Yang Hospital of Capital Medical University, Beijing, China; 5 Department of Clinical Laboratory, Peking Union Medical College Hospital, Beijing, China; 6 The First Affiliated Hospital of Medical School of Hangzhou University, Hangzhou, Zhejiang, China; 7 Department of Clinical Laboratory, The First Affiliated Hospital of Sun Yatsen University, Guangzhou, Guangdong, China; 8 Department of Clinical Laboratory, Guangdong General Hospital, Guangzhou, Guangdong, China; 9 Tongji Hospital, Tongji Medical College Huazhong University of Science & Technology, Wuhan, Hubei, China; 10 Zhongshan Hospital, Fudan University, Shanghai, China; 11 Department of Clinical Laboratory, The First Affiliated Hospital of Xi'an Jiaotong University, Xi'an, Shaanxi, China; 12 Department of Clinical Laboratory, General Hospital, Tianjin Medical University, Tianjin, China; 13 Department of Clinical Laboratory, Southwest Hospital, Chongqing, China; Fondazione IRCCS Ca' Granda Ospedale Maggiore Policlinico, Università degli Studi di Milano, Italy

## Abstract

*Streptococcus pneumoniae* is an important pathogen in both children and the elderly, but previous studies in China have provided limited information about invasive pneumococcal disease (IPD). A total of 240 IPD *S. pneumoniae* strains (from 105 children and 135 adults) were collected from 12 cities in China in 2005–2011. Their phenotypes and genetic characteristics were analyzed. *Streptococcus pneumoniae* remained highly resistant to macrolides, tetracycline, and cotrimoxazole each year. Serotypes were assigned to the 240 isolates, and 19A (22.1%), 19F (21.7%), 14 (7.5%), 3 (7.1%), and 23F (5.4%) were the most prevalent, accounting for 63.8% of all strains. Serogroup 19 strains were significantly more common among children than among adults (58.7% vs 32.4%, respectively; P < 0.001). Serotypes 19F and 19A demonstrated higher resistance to β-lactams and cephalosporins than the other serotypes. The coverage of PCV13 was superior to that calculated for PCV7 and PCV10 (77.9% vs 40.8% and 47.1%, respectively), and coverage was higher in children than in adults (85.6% vs 72.1%, respectively; P = 0.012). A multilocus sequence typing analysis revealed great diversity, with nine clonal complexes and 83 singletons among all the strains. Specifically, CC271 was more common in children, whereas singletons were more prevalent in adults. Among the serogroup 19 strains, 84.7% were ST271, ST320, or ST236, belonging to CC271. The homogeneous genetic background of 19F and 19A, together with the high resistance of these strains, suggests that clonal spread is responsible for the high prevalence of serogroup 19 in IPD. This is the first large study to investigate IPD strains in both children and adults in China.

## Introduction

*Streptococcus pneumoniae*, the leading pathogen responsible for bacterial infections in infants and the elderly, can cause various pneumococcal diseases, includes otitis media, sinusitis, pneumonia, meningitis, and bacteremia. Pneumococcal disease is a major public health problem worldwide. In 2005, the World Health Organization (WHO) estimated that 1.6 million people die of pneumococcal diseases annually, 0.7–1 million of whom are children younger than five years [1]. Invasive pneumococcal disease (IPD) is defined as the isolation of *S. pneumoniae* from a normally sterile site (e.g., blood, cerebrospinal fluid [CSF], joints, and pleural or pericardial fluid). IPD occurs most commonly in children < 5 years old and adults ≥ 65 years old.

Antibiotics are usually the primary choice of treatment for pneumococcal infections. However, the increasing resistance of *S. pneumoniae* to various antibiotics, including β-lactams, macrolides, and tetracyclines, makes it difficult to treat these infections with antibiotics in China [2, 3]. Vaccination is an alternative approach to the prevention of pneumococcal infections. Since the introduction of a heptavalent pneumococcal conjugate vaccine (PCV7), the epidemiology of *S. pneumoniae* has changed in many countries [4, 5]. One of the most prominent changes is a reduction in the prevalence of the PCV7 vaccine serotypes in immunized children, and in unimmunized adults as a result of the herd effect.

Antibiotic susceptibilities and serotype distributions in children in China have been extensively described [2, 6, 7]. PCV7 has been available in China since 2008, but only in the private market for healthy children. Previous studies have provided valuable information about pneumococcal infections but were not as comprehensive as the present study. First, all previous studies focused on children, and the epidemiology of pneumococcal infections among adults remains unknown. Second, the isolates examined in previous studies were from non-invasive sites, and limited information about invasive infections was presented in these studies. Studies of the epidemiology of invasive pneumococcal disease (IPD) in mainland China are rare because of community-level antibiotic abuse and the very low rate of *S. pneumoniae* isolation from bacterial cultures [2, 8]. In this study, we investigated the largest number of invasive strains to date and analyzed the phenotypic and genotypic characteristic of invasive pneumococcal isolates from both children and adult patients in China in the period 2005–2011. PCV13 will be introduced in China in the near future and the results of this study allow the prediction of its coverage rate.

## Materials and Methods

### Bacterial isolates

A total of 240 *S. pneumoniae* isolates were collected from invasive sites in pediatric and adult patients with pneumococcal infections in 12 cities throughout China during the years 2005–2011. Of these strains, 181 strains were from patients with bacteremia, 40 from those with meningitis, and 19 from those with pneumonia. The Ethics Committee of the Peking University People’s Hospital approved the study, and all procedures were performed in accordance with the Declaration of Helsinki (1975), as revised in 2008. All the patients were gave written informed consent to their participation in the study. Written informed consent was obtained from guardians on the behalf of the child participants (under the age of 18 years) in this study. The isolates were identified based on the typical colony morphology, Gram staining, optochin sensitivity test (Oxoid Company, Britain, Hampshire), and Omni serum assay (Statens Serum Institut, Copenhagen, Denmark). Only one isolate was selected from each subject. Of the 240 *S. pneumoniae* isolates collected, 51 were obtained from pediatric patients aged 0–2 years, 34 were obtained from pediatric patients aged 2–5 years, 20 from those aged 6–17 years, 105 from adults aged 18–64 years, and 30 from patients > 64 years old.

### In vitro antimicrobial susceptibility test

The agar dilution method was used to determine the antibiotic susceptibility of the 240 pneumococcal isolates to 12 antibiotics, according to the guidelines established by the Clinical and Laboratory Standards Institute (CLSI) [9]. The CLSI 2013 criteria [10] for minimum inhibitory concentrations (MICs) were applied to classify the susceptible, intermediate, and resistant isolates. Three separate interpretive breakpoints for nonmeningeal, meningeal, and oral isolates were used to define penicillin resistance: MICs ≥ 0.12 (meningeal) and ≥ 8 μg/ml (nonmeningeal) for parenteral penicillin, and MIC of ≥ 2 μg/ml for oral penicillin V. MICs of ≥ 2 μg/ml (meningeal) and ≥ 4 μg/ml (nonmeningeal) for ceftriaxone were used to define ceftriaxone resistance. *Streptococcus pneumoniae* strain ATCC49619 was used as the quality control strain and was included in each set of tests to ensure accurate results.

### Serotyping

Pneumococcal serotypes/groups were determined for the 240 invasive isolates with the Quellung reaction using Pneumotest kits and type-specific antisera (Statens Serum Institut, Copenhagen, Denmark), as previously described [11]. The isolates that reacted negatively were classified as “nontypable”. The potential PCV7, PCV10, and PCV13 vaccine coverages were estimated by calculating the percentage of isolates that expressed the serotypes and related serotypes included in the vaccines.

### Multilocus sequence typing (MLST) of invasive isolates

Of the 240 invasive isolates examined, 211 were investigated with MLST. Internal fragments of approximately 550–600 bp from the *aroE*, *gdh*, *gki*, *recP*, *spi*, *xpt*, and *ddl* genes were amplified by PCR using previously described primers [12]. The sequences of each of the seven loci were compared with those of all known alleles at these loci, and with the sequence types (STs) in the database of the pneumococcal MLST website (http://spneumoniae.mlst.net). Alleles not included in the present pneumococcal database were resequenced and submitted to the S. *pneumoniae* MLST database for designation. The eBURST v3 software (http://spneumoniae.mlst.net/eburst/) was used to investigate the relationships between the isolates and to assign a clonal complex (CC) based on the stringent group definition of six out of seven shared alleles.

### Statistical analysis

The data from the antibiotic susceptibility tests were set up and analyzed with the WHONET 5.6 software, a Windows-based database software developed by WHO for the management and analysis of microbiological laboratory data, with a special focus on the analysis of antimicrobial susceptibility test results. The χ^2^ test and Fisher’s exact probability test were performed in SPSS for Windows (version 18.0; SPSS, Chicago, IL) to compare proportions. Differences with *P* < 0.05 were considered statistically significant.

## Results

### Collection of pneumococcal isolates

During the study period (2005–2011), a total of 240 invasive pneumococcal isolates were collected from 12 different cities in China. Of these 240 strains, 105 were obtained from children < 5 years and 135 were obtained from adults ≥ 18 years. More strains were isolated from male patients than from female patients (159 vs 81 strains, respectively). Eighty-nine strains were from Shenyang, 47 strains from Beijing, 30 strains from Hangzhou, 26 strains from Guangzhou, 13 strains from Wuhan, 12 strains from Shanghai, and 23 strains from six other cities (Table 1). Blood was the most common specimen source, accounting for 75.4% (181 strains) of all study isolates, followed by CSF (40 strains) and pleural fluid (19 strains).

**Table 1:** Number of *Streptococcus pneumoniae* strains in different cities in the period 2005–2011.

Regions	Cities		2005	2006	2007	2008	2009	2010	2011
			Number (%)	Number (%)	Number (%)	Number (%)	Number (%)	Number (%)	Number (%)
Northeast	Shenyang		17 (60.7)	29 (49.2)	19 (30.2)	14 (36.8)	1 (7.7)	3 (14.3)	6 (33.3)
North	Beijing		1 (3.6)	7 (11.9)	19 (30.2)	6 (15.8)	5 (38.5)	6 (28.6)	3 (16.7)
	Tianjing		0 (0.0)	0 (0.0)	1 (1.6)	3 (7.9)	0 (0.0)	0 (0.0)	0 (0.0)
East	Shanghai		0 (0.0)	4 (6.8)	1 (1.6)	2 (5.3)	0 (0.0)	3 (14.3)	2 (11.1)
	Nanjing		0 (0.0)	2 (3.4)	0 (0.0)	0 (0.0)	0 (0.0)	0 (0.0)	0 (0.0)
	Hangzhou		6 (21.4)	8 (13.6)	5 (7.9)	5 (13.2)	0 (0.0)	3 (14.3)	3 (16.7)
South	Guangzhou		1 (3.6)	1 (1.7)	7 (11.1)	5 (13.2)	5 (38.5)	3 (14.3)	4 (22.2)
	Shenzheng		3 (10.7)	3 (5.1)	1 (1.6)	0 (0.0)	0 (0.0)	0 (0.0)	0 (0.0)
Southwest	Chengdu		0 (0.0)	2 (3.4)	0 (0.0)	0 (0.0)	0 (0.0)	0 (0.0)	0 (0.0)
	Chongqing		0 (0.0)	0 (0.0)	2 (3.2)	1 (2.6)	1 (7.7)	0 (0.0)	0 (0.0)
Central	Wuhan		0 (0.0)	3 (5.1)	8 (12.7)	1 (2.6)	0 (0.0)	1 (4.8)	0 (0.0)
	Xi’an		0 (0.0)	0 (0.0)	0 (0.0)	1 (2.6)	1 (7.7)	2 (9.5)	0 (0.0)
Total			28 (100)	59 (100)	63 (100)	38 (100)	13 (100)	21 (100)	18 (100)

××××××××××××××××××××××××××××××××××

### Antibiotic susceptibility

The susceptibilities of the S. *pneumoniae* strains to 12 antibiotics are summarized in Table 2. According to the revised breakpoints for parenteral penicillin, the overall prevalence of penicillin resistance was 3.8% in nonmeningeal isolates and 51.5% in meningeal isolates. During the years 2005–2011, the average rate of penicillin-nonsusceptible *S. pneumoniae* (PNSP) isolates was higher before the introduction of PCV7 in 2008 (32.4% vs 23%, respectively, for the nonmeningeal breakpoint; and 60.7% vs 33.3%, respectively, for the oral breakpoint). The susceptibilities to amoxicillin/clavulanic acid and ceftriaxone varied with the year, and ceftriaxone presented higher activities than amoxicillin/clavulanic acid. Erythromycin and clindamycin showed low potency against *S. pneumoniae* (resistance rates, > 80%). Of the erythromycin-resistant strains, 14 were susceptible to clindamycin, 10 strains were D-test positive, indicating the iMLSB phenotype, and four other D-test-negative strains showed M phenotype resistance. No resistance to vancomycin, levofloxacin, or moxifloxacin was identified during the study period. The prevalence of PRSP (nonmeningeal) was 5.9% in children < 5 years and 11.1% in those aged 6–17 years, which were higher than those in the other age groups: 18–64 years (1%) and > 64 years (3.4%). During the study period, the susceptibilities of the invasive *S. pneumoniae* strains to routine clinical antibiotics remained stable, although the susceptibility to penicillin increased from 67.8% before PCV7 to 76.9% (P = 0.41) after PCV7 in 2008 with the nonmeningeal breakpoints.

**Table 2:** Susceptibility of invasive *Streptococcus pneumoniae* to routine clinical antibiotics.

Antibiotics		2005	2006	2007	2008	2009	2010	2011
		Number (%)	Number (%)	Number (%)	Number (%)	Number (%)	Number (%)	Number (%)
Penicillin NM**^*4*^**	S^1^	25 (89.3)	34 (58.6)	39 (63.9)	26 (72.2)	8 (61.5)	14 (66.7)	18 (100.0)
	I^2^	2 (7.1)	21 (36.2)	19 (31.1)	10 (27.8)	5 (38.5)	5 (23.8)	0 (0.0)
	R^3^	1 (3.6)	3 (5.2)	3 (4.9)	0 (0.0)	0 (0.0)	2 (9.5)	0 (0.0)
Penicillin M^5^	S	11 (39.3)	23 (39.7)	30 (49.2)	21 (58.3)	7 (53.8)	10 (47.6)	12 (66.7)
	I	0 (0.0)	0 (0.0)	0 (0.0)	0 (0.0)	0 (0.0)	0 (0.0)	0 (0.0)
	R	17 (60.7)	35 (60.3)	31 (50.8)	15 (41.7)	6 (46.2)	11 (52.4)	6 (33.3)
Penicillin Oral	S	11 (39.3)	23 (39.7)	30 (49.2)	21 (58.3)	7 (53.8)	10 (47.6)	12 (66.7)
	I	10 (35.7)	7 (12.1)	4 (6.6)	2 (5.6)	0 (0.0)	2 (9.5)	2 (11.1)
	R	7 (25.0)	28 (48.3)	27 (44.3)	13 (36.1)	6 (46.2)	9 (42.9)	4 (22.2)
Amoxicillin/clavulanic	S	20 (71.4)	39 (67.2)	42 (68.9)	22 (62.9)	7 (53.8)	14 (66.7)	15 (83.3)
	I	7 (25.0)	14 (24.1)	8 (13.1)	3 (8.6)	2 (15.4)	2 (9.5)	0 (0.0)
	R	1 (3.6)	5 (8.6)	11 (18.0)	10 (28.6)	4 (30.8)	5 (23.8)	3 (16.7)
Ceftriaxone NM**^*4*^**	S	22 (78.6)	45 (77.6)	47 (77.0)	31 (88.6)	7 (53.8)	17 (81.0)	15 (83.3)
	I	4 (14.3)	7 (12.1)	9 (14.8)	0 (0.0)	3 (23.1)	1 (4.8)	1 (5.6)
	R	2 (7.1)	6 (10.3)	5 (8.2)	4 (11.4)	3 (23.1)	3 (14.3)	2 (11.1)
Ceftriaxone M^5^	S	18 (64.3)	30 (51.7)	33 (54.1)	24 (66.7)	7 (53.8)	14 (66.7)	13 (72.2)
	I	4 (14.3)	15 (25.9)	14 (23)	8 (22.2)	0 (0)	3 (14.3)	2 (11.1)
	R	6 (21.4)	13 (22.4)	14 (23)	4 (11.1)	6 (46.2)	4 (19)	3 (16.7)
Cefaclor	S	16 (57.1)	21 (36.2)	30 (49.2)	20 (58.8)	7 (53.8)	12 (57.1)	12 (66.7)
	I	0 (0.0)	6 (10.3)	1 (1.6)	1 (2.9)	0 (0.0)	0 (0.0)	0 (0.0)
	R	12 (42.9)	31 (53.4)	30 (49.2)	13 (38.2)	6 (46.2)	9 (42.9)	6 (33.3)
Erythromycin	S	2 (9.5)	0 (0.0)	2 (6.3)	2 (5.7)	1 (7.7)	0 (0.0)	2 (11.1)
	I	0 (0.0)	0 (0.0)	0 (0.0)	0 (0.0)	0 (0.0)	1 (4.8)	0 (0.0)
	R	19 (90.5)	40 (100.0)	30 (93.8)	33 (94.3)	12 (92.3)	20 (95.2)	16 (88.9)
Clindamycin	S	4 (18.2)	2 (5.0)	1 (8.3)	6 (17.1)	2 (15.4)	5 (23.8)	2 (11.1)
	I	0 (0.0)	0 (0.0)	0 (0.0)	1 (2.9)	0 (0.0)	0 (0.0)	0 (0.0)
	R	18 (81.8)	38 (95.0)	11 (91.7)	28 (80.0)	11 (84.6)	16 (76.2)	16 (88.9)
Chloramphenicol	S	14 (70.0)	27 (69.2)	25 (86.2)	31 (100.0)	9 (69.2)	16 (76.2)	15 (83.3)
	I	0 (0.0)	0 (0.0)	0 (0.0)	0 (0.0)	0 (0.0)	0 (0.0)	0 (0.0)
	R	6 (30.0)	12 (30.8)	4 (13.8)	0 (0.0)	4 (30.8)	5 (23.8)	3 (16.7)
Tetracycline	S	1 (4.8)	0 (0.0)	4 (12.5)	2 (6.7)	0 (0.0)	1 (4.8)	2 (11.1)
	I	1 (4.8)	0 (0.0)	3 (9.4)	2 (6.7)	0 (0.0)	3 (14.3)	2 (11.1)
	R	19 (90.5)	40 (100.0)	25 (78.1)	26 (86.7)	13 (100.0)	17 (81.0)	14 (77.8)
Levofloxacin	S	28 (100.0)	57 (98.3)	61 (100.0)	34 (100.0)	13 (100.0)	21 (100.0)	18 (100.0)
	I	0 (0.0)	0 (0.0)	0 (0.0)	0 (0.0)	0 (0.0)	0 (0.0)	0 (0.0)
	R	0 (0.0)	1 (1.7)	0 (0.0)	0 (0.0)	0 (0.0)	0 (0.0)	0 (0.0)
Moxifloxacin	S	28 (100.0)	56 (98.2)	61 (100.0)	35 (100.0)	13 (100.0)	21 (100.0)	18 (100.0)
	I	0 (0.0)	1 (1.8)	0 (0.0)	0 (0.0)	0 (0.0)	0 (0.0)	0 (0.0)
	R	0 (0.0)	0 (0.0)	0 (0.0)	0 (0.0)	0 (0.0)	0 (0.0)	0 (0.0)
SXT**^*6*^**	S	2 (9.1)	8 (20.0)	9 (28.1)	9 (25.7)	5 (38.5)	0 (0.0)	7 (41.2)
	I	4 (18.2)	3 (7.5)	1 (3.1)	2 (5.7)	3 (23.1)	0 (0.0)	1 (5.9)
	R	16 (72.7)	29 (72.5)	22 (68.8)	24 (68.6)	5 (38.5)	0 (0.0)	9 (52.9)
Vancomycin	S	21 (100.0)	39 (100.0)	29 (100.0)	33 (100.0)	13 (100.0)	21 (100.0)	18 (100.0)
	I	0 (0.0)	0 (0.0)	0 (0.0)	0 (0.0)	0 (0.0)	0 (0.0)	0 (0.0)
	R	0 (0.0)	0 (0.0)	0 (0.0)	0 (0.0)	0 (0.0)	0 (0.0)	0 (0.0)

^1^ S: susceptibility

^2^ I: intermediate

^3^ R: resistance

^4^ NM: Non-meningitis

^5^ M: Meningitis

^6^ SXT: Trimethoprim/Sulfamethoxazole

### Serotyping and vaccine coverage

**Figure 1:**
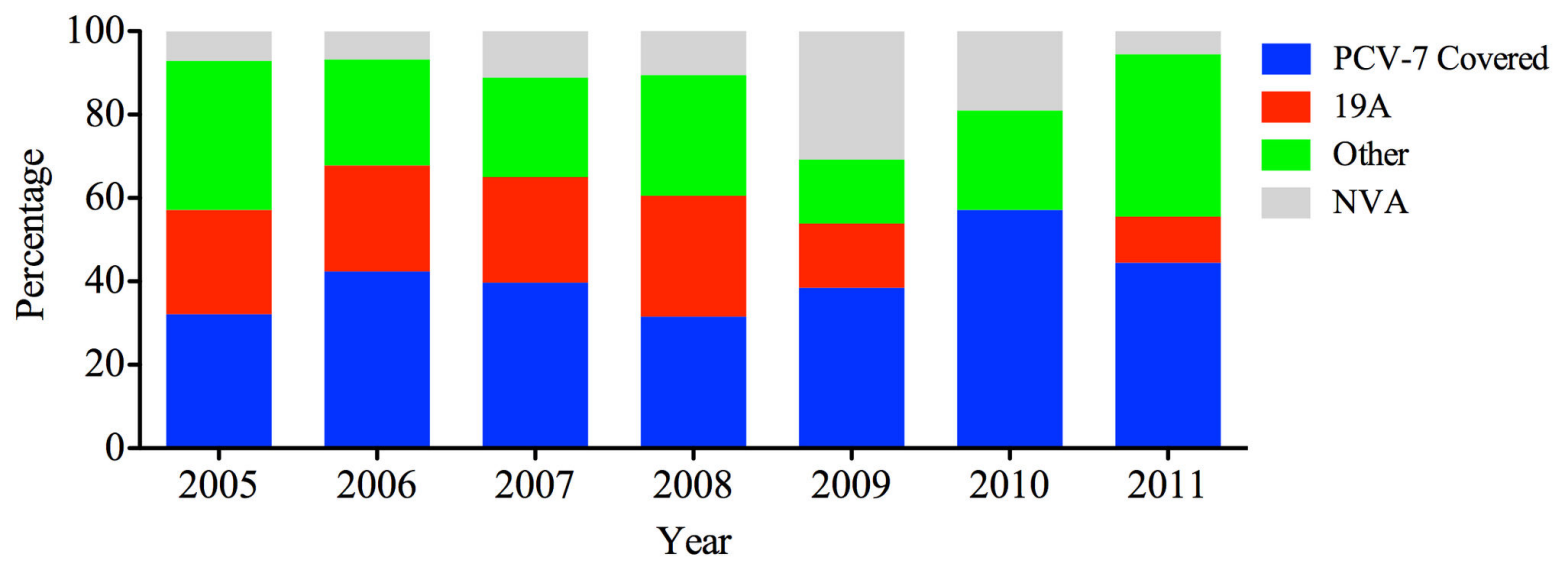
The proportion of PCV-7 and clinical important serotypes in the year 2005-2011.

Of the initial 240 invasive pneumococcus isolates, 218 (90.8%) were serotyped, whereas the remaining 22 isolates were not typable. The predominant five serotypes were 19A (53, 22.1%), 19F (52, 21.7%), 14 (18, 7.5%), 3 (17, 7.1%), and 23F (13, 5.4%), which accounted for 63.8% (153/240) of all the isolated strains. Serotype 19F strains were significantly more common among children than among adults (28.8% vs 16.2%, respectively; P = 0.018). The same trend was also observed for serotype 19A (29.8% and 16.2%, respectively; P = 0.012). There was no difference in the distribution of the other serotypes between children and adults (P > 0.05). The PCV7 and PCV10 coverages for the invasive isolates were 40.8% (98/240) and 46.3%, respectively. Although the PCV7 coverage was higher in children than in adults (45.2% and 37.5%, respectively), no significant differences in PCV7 coverage were found among the different age groups (P = 0.151). The average coverage of PCV13 for all the invasive pneumococci was 77.9%, which was significantly higher (P < 0.001) than that of PCV7 (40.8%) or PCV10 (47.1%) (Table 3). The coverage rate for PCV7 remained stable during the study period (P = 0.581), but the prevalence of serotype 19A increased after the introduction of PCV7 in 2008 (P = 0.02; Figure 1).

**Table 3:** Coverage of different vaccines against invasive *Streptococcus pneumoniae* in different age groups.

Types of Vaccine					Ages			Total
	<=5 (N=85)	6-17 (N=20)		18-64 (N=105)			>=65 (N=30)	
	Number (%)	Number (%)		Number (%)			Number (%)	Number (%)
PCV7	38 (44.7)	7 (35.0)		42 (40.0)			11 (36.7)	98 (40.8)
PCV10	41 (48.2)	10 (50.0)		51 (48.6)			11 (36.7)	113 (47.1)
PCV13	74 (87.1)	16 (80.0)		75 (69.5)			22 (73.3)	187 (77.9)

### Antimicrobial resistance and serotypes

Of the 96 invasive isolates with PCV7 serotypes, 32 were PNSP However, 66% of the serotype 19A isolates and 57.7% of the serotype 19F isolates were not susceptible to penicillin, which accounted for 91.5% of all PNSP strains. The prevalence of PNSP in serotypes 19A and 19F did not differ significantly (P = 0.249). A high prevalence of erythromycin, clindamycin, tetracycline, chloramphenicol, and co-trimoxazole resistance was observed in isolates of the PCV7 serotypes, which did not differ significantly from the resistance in the non-PCV7 strains. Serotypes 19F and 19A demonstrated higher resistance to β-lactams and cephalosporins than did the other serotypes (P < 0.001; data not shown) Serotypes 19F and 19A displayed equal resistance to penicillin, cefaclor, and cefuroxime (P > 0.05), but 19F showed higher resistance to amoxicillin and clavulanic acid (P = 0.046) and ceftriaxone (P < 0.001) than 19A (data not shown).

### MLST

**Figure 2:**
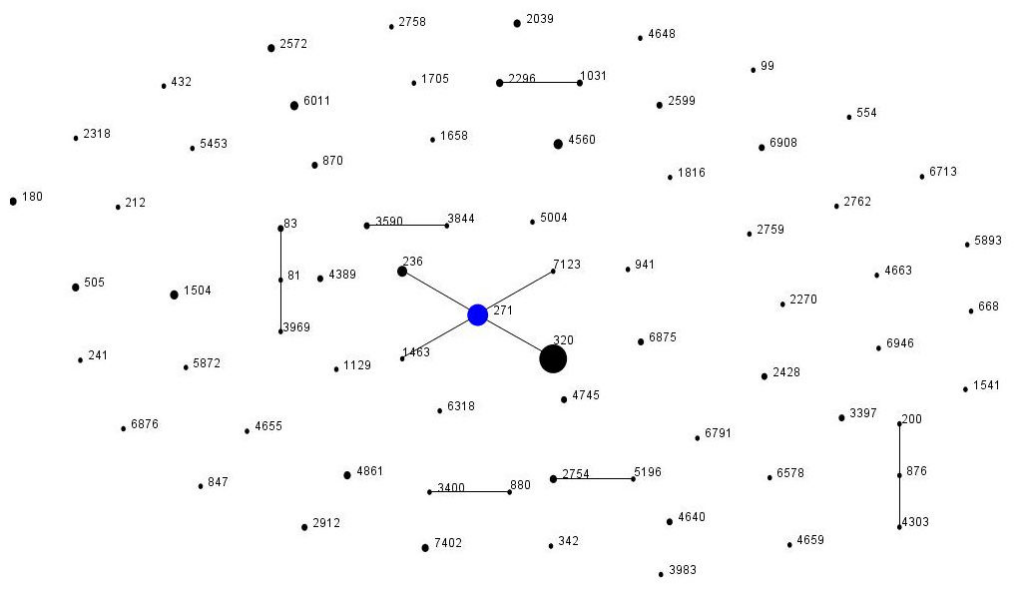
Population snapshot of 157 invasive pneumococcal isolates revealed by an eBURST analysis. The size of the dot indicates the number of strains in each sequence type. Blue dot: Founding ST. Black dot with single linkage: Single locus variant (SLV). Black dot with two linkages: Double locus variant (DLV). Black dot without linkage: Singleton.

A total of 82 STs were identified in the 240 invasive *S. pneumoniae* isolates. The four predominant STs among all the resistant pneumococci were ST320 (20.8%, 50/240), ST271 (12.9%, 31/240), ST236 (2.9%, 7/240), and ST4560 (2.1%, 5/240) (Table 4). ST320 was the ST most ubiquitously distributed among the different age groups, although the proportion of ST320 was higher in children than in adults (31.7% vs 12.5%, respectively; P < 0.001). However, the percentages of the other STs (ST236, ST271, and ST4560) did not differ between the age groups (P > 0.05). The eBURST analysis results showed nine CCs and 56 singletons (Figure 2). CC271 was the most frequent CC, comprising 40.8% (64/157) of the invasive isolates. The resistance of the ST320 strains to the penicillin nonmeningeal breakpoints was 12.0%, higher than that of ST271 (9.7%) and ST236 (0%).

**Table 4:** Sequence types, serotypes, antibiotics resistance, and age distributions for 211 invasive pneumococci analyzed by MLST.

Clonal Complex	Sequence Type	No.	Serotypes (Number)		Resistance rates of different antibiotics (%)							Number of strains in different ages			
				PEN^1^ Oral		ERY**^*2*^**		LEV**^*3*^**	≤5 (85)		6-17 (20)		18-64 (105)	≥65 (30)	
CC271	ST320	50	3(2), 19A[36], 19F[11], 23F(1)	90.0		100		0	28		5		13	4	
	ST271	31	19F[29], 19A[1], 6A(1)	87.1		100		0	13		4		11	3	
	ST236	7	19A[2], 19F[4], 23F(1)	42.9		100		0	3		1		2	1	
	ST1463	1	14(1)	100		100		0					1		
	ST6270	1	19A(1)	100		100		0					1		
	ST7123	1	N23F(1)	100		100		0					1		
CC180	ST180	4	3(3), 4(1)	0		100		0	1		1		1	1	
	ST505	3	19A[1],3(1), NVA(1)	33.3		100		0	1				2		
	ST2570	1	3(1)	0		0		0					1		
CC876	ST200	1	14(1)	0		100		0	1						
	ST876	2	14(2)	50.0		100		0					1	1	
	ST4303	1	15(1)	0		100		0	1						
CC2754	ST2754	4	14(1), 3(1), 9(1), NVA(1)	25.0		100		0	1				3		
	ST5196	2	7(1), 9(1)	0		100		0					2		
	ST7762	2	6B(2)	0		100		0					1	1	
CC81	ST81	1	19F(1)	0		100		0	1						
	ST83	2	15(1), 23F(1)	50.0				0	1				1		
	ST3969	1	23F(1)	0		100		0					1		
CC3590	ST3590	2	5(2)						1				1		
	ST3844	1	5(1)								1				
CC1031	ST1031	2	5(2)	0		50.0		0					2		
	ST2296	4	1(4)	0		100		0			1		3		
CC3400	ST880	1	23F(1)	100		100		0			1				
	ST3400	1	19F(1)	100		100		0					1		
CC2758	ST2758	1	9(1)	0		100		0					1		
	ST7402	1	9(1)	0		100		0					1		
Singletons	4560	5	6B[1], 14(1), NVA(3)	0		100		0					3	2	
	1504	4	2(3), 5(1)	0		50		0	2				2		
	6011	4	15(3), 3(1)	0		100		0					2	2	
	2572	3	19F[1], 23F[1], 7(1)	0		100		0					2	1	
	4861	3	19A(3)	0		100		0	1				1	1	
	2039	3	NVA(3)	0		100		0	2				1		
	3397	2	15(1), 23F(1)	50		100		0	1		1				
	6908	2	17(1), NVA(1)	0		100		0					1	1	
	4389	2	10(1), 3(1)	0		100		0					1	1	
	2912	2	19F[1], 6A(1)	0		0		0	2						
	Others	53	NVA[13], 14(7), 19A[4], 3(4), 11(3), 23F(3), 15(2), 18(2), 19F(2), 9(2), 17(1), 2(1), 20(1), 22(1), 33(1),4(1), 5(1), 6A(1), 6B[1],8(1),N23F(1)	3.8		88.1		1.9	13		3		28	9	

^1^ PEN: Penicillin

^2^ ERY: Erythromycin

^3^ LEV: Levofloxacin

## Discussion

*Streptococcus pneumoniae* is an extremely important human bacterial pathogen, and is associated with a high rate of mortality, especially in young children, older adults, and immunocompromised patients. IPD is a disease that mainly occurs in children aged < 5 years and adults aged ≥ 65 years. Individuals with severe chronic disease or immunodeficiency are also at increased risk of IPD [13]. This study describes the changing trends in the antimicrobial resistance and serotype distributions of invasive pneumococcal isolates in both children and adults, collected from 10 Chinese cities during 2005–2011.

When we considered the changing trends in antimicrobial resistance in China, the first remarkable finding was the persistent high level of macrolide resistance, which is consistent with another report [3]. Previous studies of clinical isolates and nasopharyngeal isolates have already shown that many Chinese cities have a much higher prevalence of macrolide resistance among pneumococci than the Western countries [2, 3, 14]. More seriously, the MIC_90_ of macrolides was 256 μg/ml. The major reasons for the high level of macrolide resistance in Chinese cities are the widespread use of macrolides in clinical practice and the clonal spread of macrolide-resistant strains. Given the current epidemiology of macrolide resistance, the empirical use of macrolides alone for the treatment of pneumococcal infections may be an inappropriate choice in China, where it may cause a failure of this antimicrobial therapy. The rates of resistance and insensitivity to β-lactams and cephalosporins also show upward trends. Fluoroquinolones and glycopeptides retain good activities against invasive *S. pneumoniae*, and could be used as alternative treatments for infections caused by resistant strains in adults. Amoxicillin/clavulanic acid could be the primary choice for the treatment of pneumococcal infections in children at the risk that up to 20% of the resistance.

Our serotype distribution analysis showed that serotypes 19A, 19F, 3, 14, and 23F were the most frequent, of which only 19A is not included in PCV7. The overall coverage rate of PCV7 was 40.0% in the present study. This rate is much lower than that reported for the serotypes associated with IPD in young children in developed countries before the introduction of PCV7 (65%–80%) [1]. The composition of the PCV7 vaccine was initially designed to protect children against the serotypes that commonly cause disease in North America, and may not reflect the serotype distributions in China and surrounding areas [15]. According to the data from this study, PCV7 and PCV10 have similar coverage rates against the invasive strains, because the extra serotypes included in PCV10 (1, 5, and 7F) are rarely detected. However, PCV13 would increase the coverage by 30%, mainly because of the high prevalence of serotype 19A. PCV13 is likely to prevent more cases of pneumococcal disease in China because the incidence of 19A is high, suggesting that PCV13 should be used in the future in China. The vaccine shows different efficacy in different age groups. The coverage rates in children < 5 years old are much higher than those in the older age groups because the immaturity of the newborn immune system constitutes physiological immunodeficiency and increases the susceptibility of young children to infections by *S. pneumoniae* [16].

Several studies have shown that the mass-scale use of PCV7 resulted in the spread of type 19A *S. pneumoniae* and reduced the rate of type 19F [17-19]. PCV7 was licensed and introduced into China in 2008. However, the data from this study indicate that the same phenomenon is not observed in China, where serotype 19A was prevalent in IPD even before the introduction of PCV7. In the present study, it is noteworthy that isolates of serotype 19A, which is not included in PCV7, are prevalent in China, with high levels of antibiotic resistance. Specifically, serotype 19A was prevalent in IPD before the introduction of PCV7, but decreased significantly after 2008. The major reason for the reduced rate of 19A was the changed composition of the strain population in Shenyang, because strains from Shenyang accounted for 37.1% of all the IPD strains in this study. Among the total serogroup 19 strains, the proportion from Shenyang dropped from 57.1% in 2005–2006 to 11.1% in 2009–2011, and the rate of 19A in Shenyang decreased significantly from 77.3% in 2005–2006 to 0% in 2009–2011. However, because the number of IPD strains collected in Shenyang decreased from 46 to 10 over the same period, the frequency data for the serogroup 19 strains my not be reliable. We must analyze the noninvasive strains from the same site to clarify whether the invasiveness of serogroup 19 has diminished.

The MLST analysis revealed a great diversity among all the IPD strains in terms of their STs, but all the resistant strains were identified as ST271, ST320, and ST236, which belong to the same clonal complex, CC271. Because of the high levels of resistance among the serotype 19A isolates and their homogeneous genetic background, it is conceivable that the clonal spread of type 19A, before the introduction of PCV7, was caused by the selective pressure imposed by antibiotic abuse. A study undertaken in South Korea demonstrated a similar phenomenon in which the expansion of multidrug-resistant ST320 was responsible for an increase in serotype 19A before the introduction of PCV7 [20]. Moreover, surveys in Western countries that have examined the long-term effects of selective pressure imposed by PCV7 have reported that non-PCV7 serotypes, and 19A in particular, have become not only more prevalent, but also more resistant to antimicrobial drugs [21-23]. These findings suggest that PCV7 vaccinations may not be solely responsible for the observed increase in serotype 19A where PCV7 is widely used, and that antimicrobial abuse could also play an important role in the serotype replacement. In this study, serotype 19A and serotype 19F displayed equal resistance to penicillin and other routine clinical antibiotics. These findings suggest that PCV7 vaccination may not be entirely responsible for the observed increase in serotype 19A in areas where PCV7 is widely used, and that antimicrobial abuse could also play an important role in the high prevalence of serogroup 19. These results suggest that rational antimicrobial use must be implemented to control the emergence of resistance and preserve the preventive effectiveness of the vaccine in the long term when PCV7 is the primary vaccine, before PCV13 is introduced.

This is the first study to investigate invasive *S. pneumoniae* in both children and adults, and the population of invasive strains examined was much larger than in other studies in China. However, the study has several limitations. First, the number of invasive isolates varied with years and locations. It was impossible to preclude sampling bias because *S. pneumoniae* strains from invasive sites are so rare in China. Second, all the strains were isolated from hospitalized patients and cannot provide adequate information on community infections. Third, the study was performed in several relatively developed cities in China and does not represent the situation in the whole of China, especially in rural and remote areas. In summary, the characteristics of drug resistance and serotype distribution revealed in this study suggest that a more effective vaccine should be used to prevent pneumococcal infections caused by resistant serotypes, particularly serotype 19A. PCV13 is likely to prevent more episodes of pneumococcal disease in China than other vaccines because the rates of 19A are high. Long-term surveillance is required to monitor antimicrobial resistance and serotype distributions.
